# Residential density was negatively associated with excess body weight among adults in an urban region of China

**DOI:** 10.1371/journal.pone.0219314

**Published:** 2019-07-10

**Authors:** Na Wang, Zhiyong Wang, Zhenzhen Qin, Qing Ye, Peng Jia, Zhen Xu, Yaqing Xiong, Fei Xu

**Affiliations:** 1 Nanjing Municipal Center for Disease Control and Prevention, Nanjing, China; 2 GeoHealth Initiative, Department of Earth Observation Science, Faculty of Geo-Information Science and Earth Observation (ITC), University of Twente, Enschede, The Netherlands; 3 International Initiative on Spatial Lifecourse Epidemiology (ISLE), Enschede, The Netherlands; 4 College of Landscape Architecture, Nanjing Forestry University, Nanjing, China; 5 Jiangsu Province Official Hospital, Nanjing, China; 6 School of Public Health, Nanjing Medical University, Nanjing, China; McMaster University, CANADA

## Abstract

**Background:**

Residential density was found to be associated with excess body weight among adults in Western societies but it remains unclear in China. We aimed to explore the relationship between residential density and excess body weight among adults in China.

**Methods:**

This cross-sectional study was conducted in 2017 in urban areas of Nanjing, China. A multi-stage sampling method was used to randomly select participants aged 35–74 years from 8 urban neighborhoods in Nanjing. Status of excess body weight was the outcome variable which was categorized as "yes (BMI≥24)" or "no (BMI<24)" according to specific recommendations for Chinese adults. Residential density was the main explanatory variable which was grouped into tertiles. Odds ratios (OR) and 95% confidence interval (CIs) were calculated to assess the relationship between residential density and excess body weight using mixed-effects regression models after adjusting for age, sex, education, occupation, marital status, redmeat, smoking, physical activity, diabetic status and potential neighborhood-level clustering effect.

**Results:**

A total of 1551 participants were recruited with a response rate of 98.9% (1551/1568). The mean age (standard deviation) of participants was 54.7 (11.1) years, and 46% of them were men. With adjustment for potential influential factors, participants in neighborhoods with lower (OR = 1.38, 95% CI = 1.06–1.81) and middle (OR = 1. 29, 95% CI = 1. 01, 1. 64) tertile of residential density were at significantly higher risk of gaining excess body weight relative to their counterparts in communities with upper tertile of residential density. Such a negative association between residential density and excess body weight was observed for men only after stratified analysis by gender.

**Conclusions:**

A negative association between residential density and excess body weight was identified in overall and male urban Chinese adults, and the association was independent of physical activity. Results of our study have important implications in guiding public health policy making regarding prevention of excess body weight at community level via establishment of health-friendly neighborhood environment in China.

## Introduction

Excess body weight (EBW), referring to overweight or obesity, has been becoming a serious public health problem worldwide, including in China [1]. EBW, as an unhealthy lifestyle related chronic condition, can also lead to several health consequences, such as metabolic syndromes, hypertension and cardio-/cerebro-vascular diseases [2, 3]. Moreover, the prevalence of EBW has been rising sharply worldwide over past several decades [4]. Therefore, it is of significant importance for EBW prevention to identify amendable EBW risk factors at community level in a cost-benefit basis.

EBW is related to adverse energy imbalance that mainly results from more energy intake than energy expenditure for human body. Consumption of high-energy foods and lack of physical activity (PA) are the major established lifestyle-related contributors of EBW [1, 5]. Recently, growing evidence suggests a link between neighborhood built environment (BE) and EBW [6, 7]. Built environment refers to all buildings, spaces and products that are created or modified by people [8], which includes several domains (urban design, transportation systems, land-use and policies, etc) [6, 9]. Residential density (RD) is an indicator of built environment and usually used to assess urban design and land-use regarding built environment attributes. RD is a measure of units within a specific geographic area to reflect the density of population or households [9, 10, 11]. In this study, RD specifically means population density of neighborhoods. RD implies inter-correlation with other built environment attributes and is more comprehensive and sensitive than other built environment measures [12, 13, 14, 15].

To date, all previous studies regarding RD-EBW association among adults used the cross-sectional design and were mainly conducted in Western communities, but the findings were inconsistent [16–25]. Most of these previous studies documented that RD was negatively associated with EBW [16–23], while the others reported non-significant results [24, 25]. For China, the relationship between RD and EBW among adolescents in urban areas has been studied [26], but its relation in urban adults is not known. Thus, further studies are needed to explore the relationship between RD and EBW worldwide, including developing societies like China where economic growth and urbanization have been leading to neighborhood construction. With the rapid economic development and urbanization over the past decades, China has been witnessing substantial changes in city planning and neighborhood construction. On one hand, the geographic size of urban areas in large cities of China has been expanding; on the other hand, the per capita living space of urban residents is becoming more crowded and denser. For example, the geographic size of urban areas in our study city, Nanjing, increased by 39.5%, from 186.7 km^2^ in 2006 to 260.5 km^2^ in 2016. However, RD in the areas still increased from 8533 to 9267 persons/km^2^ during the time period [27]. Therefore, we conducted this study with attempt: (1) to examine the relationship between RD and EBW among overall urban adults in regional China, and (2) then to compare the gender difference in such RD-EBW association.

## Methods

### Participants and sample selection

This population-based cross-sectional study, namely The Built Environment and Chronic Health Conditions: Adults (BEACH-Adults), was conducted during the period of March and July of 2017 in urban areas of Nanjing (regular inhabitants: >8 million), one of the typical mega-cities in China. Nanjing had six urban and five suburban administrative districts in 2016 when this study was designed and proposed. The urbanization rate was 82.0% in Nanjing in 2016 [28], much higher than the average level (57.4%) of China in the same year [29].

Our BEACH-Adults study was originally developed: to (1) validate the Chinese version of Physical Activity Neighborhoods Environment Scale (PANES-CHN); and (2) to investigate relationship between neighborhood built environment attributes and chronic health conditions (including physical activity). We considered the participant’s inclusion eligibility and sampling approach mainly based on the requirement for validating the Chinese version of Physical Activity Neighborhood Environment Scale (PANES-CHN) [30]. In China, chronic health conditions (including obesity) were usually investigated among people aged ≥35 years old. So we selected the study participants using the age cutoffs of ≥35 years old in our study. Our sample size estimation and sampling approach have been described in detail elsewhere [30]. In brief, the eligible participants were the adults aged 35–74 years old, who lived in urban districts of Nanjing, and had no physical disability. We estimated the sample size using cross-sectional study design and the recruitment of approximate 1600 participants was regarded as having adequate statistical power [30]. A multi-stage sampling method was applied to randomly select our participants. Firstly, two urban districts were randomly selected from all the six urban areas. Then, four neighborhoods were randomly selected from each chosen district. Thus, eight neighborhoods in total were our survey communities in this study. Finally, approximately 200 adults were randomly selected from each neighborhood, with frequency matching in age (8 sub-groups with 5-year interval) and gender (men *vs*. women = 1 *vs*. 1 within each age-group). Such a sampling approach could maximize our sample’s representativeness of general population.

Each participant was asked to provide a written informed consent prior to our survey. This study was approved by The Academic and Ethical Committee of Nanjing Municipal Center for Disease Control and Prevention.

### Data collection

In our BEACH-Adults study, face-to-face interview was carried out by our trained research staff to collect information based on a standardized 4-page questionnaire which was widely used in population-based epidemiological surveys in China [31]. The questionnaire contains information on participants’ demographic characteristics, personal chronic disease history (health status) and lifestyle/behavior, which could be completed in 15–20 minutes. In the survey, diet intake was assessed using weekly frequency of intake and all the diet-related items used in our study were adopted from a validated Chinese version of food frequency questionnaire (FFQ) [32]. Built environment features were extracted from the validated PANES-CHN [30], while PA level was recorded using the validated Chinese version of International Physical Activity Questionnaire (IPAQ-CHN) [33]. No personal identification was included in the analyzed dataset.

### Measures and variables

#### Outcome variable

Body weight status was the outcome variable in this study, which was assessed with body mass index (BMI) and classified into two sub-groups: excess body weight (BMI>24) or non-excess body weight (BMI<24) [34]. The cut-offs used to define excess body weight in this study was officially recommended by Ministry of Health of China for Chinese adult people [34]. We asked the participants to wear light clothing and without shoes when measuring their body weight and height. Body weight was measured to the nearest 0.1 kg using a beam balance scale, and height was measured to the nearest 0.01 meter using a stadiometer. Each of these two anthropometric measures was assessed twice and the mean value was used in our analysis.

#### Independent variable

RD was the independent variable in the study, which was defined as the number of regular habitants divided by geographic area size (km^2^) of each study neighborhood. RD was classified into three sub-groups according to tertile distribution (<29.8, 29.8–56.5, and > = 56.5 thousands person/km^2^). All the participants within a neighborhood shared the same value of RD. Information on number of regular residents and area size for each neighborhood were collected from local bureau of statistics [28, 35].

#### Covariates

In our analysis, participants’ socio-demographic characteristics, the identified lifestyle and behavioral factors (redmeat intake, physical activity and smoking) for excess body weight and participants’ diabetic status were considered as covariates. The selected socio-demographic characteristics included age (35–49, 50–64 or 65–74 years old), sex (men or women), marital status (married or others), educational attainment (≤9, 10–12 or 13+years) and occupation (while collar or blue collar). It has been reported that redmeat intake and smoking were associated with excess body weight in China [36,37], thus we put redmeat intake (dichotomized as “upper” or “lower” based on median of weekly consumption frequency) and smoking status (“yes” or “no”) into consideration in our analysis. Current smokers referred to those who smoked at least one cigarette per day continuously for at least one year, or at least eighteen packs in the previous year [38]. Additionally, as one of the main behavior-related influence factors of EBW, physical activity was also considered in the analysis. Participants were classified as having "sufficient PA" or "insufficient PA" according to sum of the last 7-day’s moderate PA time plus doubled vigorous PA (cutoff value: 150 minutes/week) time based on recommendation by USA Department of Health and Human Services [39]. Diabetic status was self-reported by participants and categorized as “yes” or “no” in our analysis.

### Data analysis

We conducted variance analysis for continuous variables or χ^2^ tests for categorical data to make comparison of participants’socio-demographic characteristics within gender and RD categories, separately. Mixed-effects models were introduced to compute odds ratio and 95% confidence interval (OR and 95%CI) to assess the RD-EBW association in this study. To look at the influence of potential confounders on the association between RD and EBW, we introduced two models: Model 1 was an unadjusted model with RD as the independent variable; Model 2 was a multilevel analysis with consideration of participants’ age, sex, education level, occupation, marital status, redmeat intake, smoking status, physical activity and diabetic status. Potential neighborhood-level clustering effect was also controlled for with neighborhood as the random effect in our mixed-effects analysis. Data were entered using EpiData 3.1 (The Epidata Association, Odense, Denmark) and analyzed with SPSS 21.0 (IBM Corp, Armonk, NY, USA).

## Results

A total of 1568 people were invited to participate in our study, and 1551 (98.9%) of them were successfully interviewed. Participants’ selected socio-demographic characteristics are shown in Table 1. The mean (Standard Deviation) age of all participants were 54.7 (11.1) years, and 22.7% of them obtained at least 13 years of educational attainment. Less than one-third of all participants (28.5%) were engaged in sufficient PA and 51.3% of them had excess body weight. There was no statistical significance in age distribution among RD subgroups; however, participants who received educational attainment of less than 9 years, had excess body weight or sufficient PA tended to live in neighborhoods with lower level of RD.

**Table 1:** Selected characteristics of participants by gender and residential density in 2017 in Nanjing, China ^a^.

	Overall	Gender		*P value*	Residential density ^b^			*P value*
		Men	Women		Lower	Middle	Upper	
Number	1551	47.8 (741)	52.2 (810)		27.2 (422)	34.3 (532)	38.5 (597)	
Age (years, Mean±SD)	54.7±11.1	54.8±11.4	54.7±10.9	0.97	55.8±10.8	54.4±10.9	54.3±11.5	0.08
Age group (years)								
35–49 (younger)	35.9 (557)	36.8 (273)	35.1 (284)	0.10	31.0 (131)	36.7 (195)	38.7 (231)	0.07
50–64 (middle-aged)	42.0 (652)	39.4 (292)	44.4 (360)		44.1 (186)	43.6 (232)	39.2(234)	
65+ (elder)	22.1 (342)	23.8 (176)	20.5 (166)		24.9 (105)	19.7 (105)	22.1 (132)	
Educational attainment (years)								
≤9	47.3 (733)	40.2 (298)	53.7 (435)	<0.01	65.5 (277)	44.4 (236)	36.9 (220)	<0.01
10–12	30.0 (466)	32.4 (240)	27.9 (226)		21.8 (92)	34.4 (183)	32.0 (191)	
≥13	22.7 (352)	27.4 (203)	18.4 (149)		12.6 (53)	21.2 (113)	31.2 (186)	
Sufficient PA^c^								
No	71.5 (1109)	71.8 (532)	71.2(577)	0.82	63.3 (267)	67.7 (360)	80.7 (482)	<0.01
Yes	28.5 (442)	28.2 (209)	28.8 (233)		36.7 (155)	32.3 (172)	19.3 (115)	
BMI Category (kg/m^2^) ^d^								
<24	48.7 (755)	45.5 (337)	51.6 (418)	<0.01	42.9 (181)	47.2 (251)	54.1 (323)	<0.01
24+	51.3 (796)	54.5 (404)	48.4 (392)		57.1 (241)	52.8 (281)	45.9 (274)	

^a^ Results presented as mean ± standard deviation for continuous data, and as percentages for categorical data.

^b^ Residential density tertile cut-off values are 56,524 and 29,786 persons/km^2^.

^c^ Participants were classified as having "sufficient PA" or "insufficient PA" according to the total time of last 7-day’s moderate PA plus doubled vigorous PA (cutoff value: 150 minutes/week).

^d^ Cutoffs for BMI was based on recommendation for Chinese adults. BMI = 24+ was defined as excess body weight.

Overall and gender-stratified associations between residential density and excess body weight are presented in Table 2. Compared with the upper RD subgroup, the crude OR for participants in the lower and middle RD subgroups were 1.57 (95%CI = 1.22, 2.01) and 1.32 (95%CI = 1.04, 1.67), respectively (Model 1). After adjusting for potential confounding factors and neighborhood-level clustering effects, the negative relationship between residential density and excess body weight still remained statistically significant although it was slightly attenuated (Model 2). However, such a negative gradient association between residential density and excess body weight was not observed for women when data were analyzed for men and women, separately.

**Table 2:** Associations of excess body weight with residential density (OR and 95%CI) among urban men and women in 2017 in Nanjing, China.

Residential density ^a^	Participants having excess body weight (% and n/N) ^b^	Mixed-effects logistic regression models			
		Model 1 ^c^		Model 2 ^d^	
		OR (95% CI)	*P value*	OR (95% CI)	*P value*
Overall					
Upper	45.9 (274/597)	1.00		1.00	
Middle	52.8 (281/532)	1.32 (1.04–1.67)	0.02	1.29 (1.01–1.64)	0.04
Lower	57.1 (241/422)	1.57 (1.22–2.02)	<0.01	1.38 (1.06–181)	0.02
Men					
Upper	49.0 (142/290)	1.00		1.00	
Middle	57.4 (143/249)	1.41 (1.00–1.98)	0.05	1.38 (0.97–1.97)	0.08
Lower	58.9 (119/202)	1.49 (1.04–2.15)	0.03	1.48 (1.01–2.19)	0.04
Women					
Upper	43.0 (132/307)	1.00		1.00	
Middle	48.8 (138/283)	1.26 (0.91–1.75)	0.16	1.26 (0.89–1.78)	0.20
Lower	55.5 (122/220)	1.65 (1.16–2.34)	0.01	1.28 (0.88–1.88)	0.20

n: number of participants within higher physical activity category; N: total number of participants within sub-group of residential density.

^a^ Residential density was analyzed as a trichotomous variable (Lower, Middle and Upper tertile, with cut-off values of 56,524 and 29,786 persons/km^2^).

^b^ physical activity was analyzed as a dichotomous variable (≥150mins/week vs.<150mins/week).

^c^ Model 1 was a univariate analysis with residential density as the single predictor.

^d^ Model 2 was a multivariate mixed-effects logistic regression model with adjustment for age, sex (overall model only), marital status, educational attainment, occupation, smoking status, redmeat intake, physical activity, diabetic status and potential neighborhood-level clustering effects.

## Discussion

In this population-based survey conducted within regional China, we aimed to investigate the relationship between RD and EBW among overall urban adults and then to look at the difference in the potential RD-EBW association between men and women in China. We found that residential density was negatively associated excess body weight among adults in a rapidly urbanizing area of China. For overall participants, those adults within neighborhoods with residential density of lower and middle tertile, respectively, had 1.38-fold and 1.29-fold higher likelihood to have excess body weight relative to their counterparts in upper tertile residential density areas, after consideration of potential confounding factors. However such an association was restricted to male participants.

Our findings were consistent with those from the Western societies [16–25]. For Western communities, the majority of available studies documented a negative relationship between residential density and overweight/obesity among adults [16–23], while very few found no significant association between them [24–25]. One of the widely acknowledged mechanisms to explain the negative RD-EBW association was that people lived in highly population-densed communities might be more likely to engage in physical activity [40–44]. Highly population-densed neighborhoods usually have lots of easily accessible destinations, e.g., shops, restaurants, recreational facilities, which may encourage local residents to have physically active regular lives [15]. This might give an explanation on the negative relationship between residential density and overweight/obesity in Western societies. However, the association between residential density and physical activity in this study was different from those findings in Western communities. In present study, people in neighborhoods with upper (OR = 0.31, 95% CI = 0.21, 0.47) and middle (OR = 0.70, 95% CI = 0.05, 0.99) tertile of residential density was, by gradient, less likely to achieve sufficient physical activity compared to their counterparts in communities with lower residential density. Therefore, physical activity might not be able to explain the negative relationship between residential density and excess body weight for urban adults in our study city.

In addition to physical activity, energy-densed foods might be another important contributing factor to the excess weight gain, which is caused by sustained positive energy balance in that energy intake is consistently greater than its expenditure. Residents’ energy intake might be a mediator for the relationship between residential density and excess body weight in this study. However, due to lack of data on participants’ energy intake, we were not able to do analysis to address this issue. Future well-designed studies are desired to investigate the association between residential density and body weight status after taking consideration of potential mediators including physical activity and energy intake.

Although the overall urbanization rate was not high in China, the speed of urbanization was really fast over past decades. There was a 3.2-fold increase in national average urbanization rate from 1978 (17.9%) to 2016 (57.4%) for China [29]. Further, considering that China has a huge number of population, residential density in urban areas may be tremendously high. For example, the residential density in our study city, Nanjing, was 9267 persons/km^2^ in 2016 [28, 35], which is much higher than that in cities of Western societies [16–25]. Meanwhile, the definition and categories of residential density, and the study independent variable of our study were also different from those used in studies conducted in Western communities. Residential density was generally dichotomized as "high" or "low" with the cutoff of ≥ 500 persons/km^2^ in studies of Western societies, and this RD level was relatively lower than that of our study city[45]. It is not likely to adopt the same definition and category of residential density in China as that in the Western societies because no urban areas would be classified into "low density" category if the cutoff point of RD (500 persons/km^2^) used in the Western societies was adopted in China. In our survey, residential density was tertiled into three sub-groups so that we could reliably use information to examine the relationship between residential density and excess body weight, and further explored the potentially gradient association.

Interestingly, the findings of our study (conducted in 2017) among adults were not in line with that of the previous survey (conducted in 2004) among school students in urban areas of Nanjing, in which a significantly positive relationship between residential density and excess body weight was observed among Chinese adolescents [26]. The disparities between adults and adolescents should not be explained by misclassification of RD and EBW, because the same criteria of these variables were adopted in these two studies and they were conducted in the same city (Nanjing) of China. However, contributing factors to the residential density and excess body weight among adolescents may differ from those of the adults. Our finding thus has great public health implications to encourage further studies to explore the mechanisms behind the relationship between residential density (including other built environment attributes) and excess body weight for adults and adolescents in the context of China, the most populous community with rapid urbanization in the world.

The strengths of this study should be mentioned. First, this is the first study investigating the association between residential density and excess body weight among adults in China. Second, in the present study, residential density was categorized into tertiles not binary sub-groups, so we were able to explore a gradient relation between residential density and excess body weight. Third, body weight and height were objectively measured twice and the mean value of the readings was used to calculate the BMI for each participant. Finally, participants’ socio-demographic characteristics, smoking status, redmeat intake, physical activity, diabetic status and potential neighborhood-level clustering effect were controlled in our analysis.

Despite of the strengths, there were also some limitations for this study. First, the cross-sectional data of this study could not allow us inferring any causal relationship between residential density and excess body weight. Second, energy intake, an important influence factor for excess body weight, was not fully considered in this study due to unavailability of data, although redmeat consumption was adjusted for in the data analysis. Third, residential density is a macro-level indicator of built environment attribute, which has some inter-correlation with other markers of built environment attribute, e.g., street intersection. Fourth, participants were limited to those aged 35–74 years old, so it would be prudent to extrapolate the findings to local residents with different age. Finally, the statistical power might still be a concern, as only eight neighborhoods were involved. Although a multi-stage randomly sampling approach was applied and neighborhood-level clustering effects were considered in the analysis, the relation might be diluted.

Neighborhood built environment could have long-term and sustainable influences on behaviors for people who live in the neighborhood once it was constructed [46]. Therefore, it is important for health professionals and city planners to identify those health-favorable built environment attributes and put them into consideration when designing city plan. Our study revealed that residential density might exert influence on people’s body weight, implying that neighborhood residential density should be considered for fighting against excess body weight through neighborhoods planning and construction in China where rapid urbanization is happening. This might be a different aspect for prevention of excess body weight at community level.

## Conclusions

A negative association between residential density and excess body weight was observed among overall and male urban adults in regional China. It is of great public health importance to identify the specific built environment features in different communities so as to inform policy-making and city-planning for prevention of excess body weight at population level.
